# Quantitative PCR-based genome size estimation of the astigmatid mites *Sarcoptes scabiei, Psoroptes ovis *and *Dermatophagoides pteronyssinus*

**DOI:** 10.1186/1756-3305-5-3

**Published:** 2012-01-04

**Authors:** Kate E Mounsey, Charlene Willis, Stewart TG Burgess, Deborah C Holt, James McCarthy, Katja Fischer

**Affiliations:** 1 Infectious Diseases Division, Queensland Institute of Medical Research, PO Royal Brisbane Hospital, QLD, 4029 Australia; 2 School of Health and Sports Science, University of the Sunshine Coast, Maroochydore DC, QLD, 4558 Australia; 3 Parasitology Division, Moredun Research Institute, Pentlands Science Park, Bush Loan, Edinburgh, Midlothian EH26 0PZ, Scotland, UK; 4 Menzies School of Health Research, Charles Darwin University, PO Box 41096, Casuarina NT, 0810 Australia; 5 School of Medicine, University of Queensland, Herston QLD, 4005 Australia

**Keywords:** *Sarcoptes scabiei*, *Psoroptes ovis*, *Dermatophagoides pteronyssinus*, genome size

## Abstract

**Background:**

The lack of genomic data available for mites limits our understanding of their biology. Evolving high-throughput sequencing technologies promise to deliver rapid advances in this area, however, estimates of genome size are initially required to ensure sufficient coverage.

**Methods:**

Quantitative real-time PCR was used to estimate the genome sizes of the burrowing ectoparasitic mite *Sarcoptes scabiei*, the non-burrowing ectoparasitic mite *Psoroptes ovis*, and the free-living house dust mite *Dermatophagoides pteronyssinus*. Additionally, the chromosome number of *S. scabiei *was determined by chromosomal spreads of embryonic cells derived from single eggs.

**Results:**

*S. scabiei *cells were shown to contain 17 or 18 small (< 2 μM) chromosomes, suggesting an XO sex-determination mechanism. The average estimated genome sizes of *S. scabiei *and *P. ovis *were 96 (± 7) Mb and 86 (± 2) Mb respectively, among the smallest arthropod genomes reported to date. The *D. pteronyssinus *genome was estimated to be larger than its parasitic counterparts, at 151 Mb in female mites and 218 Mb in male mites.

**Conclusions:**

This data provides a starting point for understanding the genetic organisation and evolution of these astigmatid mites, informing future sequencing projects. A comparitive genomic approach including these three closely related mites is likely to reveal key insights on mite biology, parasitic adaptations and immune evasion.

## Background

There is a distinct paucity of genomic data available for the class Acari (mites and ticks), and subsequently our understanding of their biology is limited. More information regarding mite genomics would greatly assist the development of novel control strategies underpinned by molecular approaches. Three astigmatid mites of particular medical and veterinary interest are the itch mite *Sarcoptes scabiei *(Sarcoptidae), the sheep scab mite *Psoroptes ovis *(Psoroptidae) and the house dust mite *Dermatophagoides pteronyssinus *(Pyroglyphidae).

Scabies remains a truly neglected disease, caused by the burrowing ectoparasitc "itch mite" *S. scabiei*. The scarcity of molecular data on *S. scabiei *has been due in part to very low parasite burden in most patients, and the historical lack of *in vitro *culture and animal models. A major advance was the creation of *S. scabiei *var. *hominis *cDNA libraries resulting in a database of ~43,000 Expressed Sequence Tags (ESTs), providing substantial molecular data for this parasite and securing a solid base for recombinant biology [1,2]. More recently, a tractable porcine model of scabies has been established [3], providing large amounts of mite material for molecular studies. *P. ovis *is a non-burrowing, ectoparastic mite causing the highly infectious disease 'sheep scab' in sheep and cattle, responsible for major economic losses and serious welfare concerns [4]. There is a similar dearth of understanding regarding parasite biology, but recently over 1,500 ESTs have been generated and deposited in public databases, representing the largest molecular data resource on *P. ovis *to date [5]. Finally, although allergies to house-dust mites are extremely common, remarkably little genetic information is available on the causative agents, the free-living mites *D. pteronyssinus*, *D. farinae *and *Euroglyphus maynei*. Research efforts have mainly focused on characterisation and generation of recombinant house dust mite allergens for diagnostics and immunotherapy [6]. However, a dataset of ~3000 ESTs is available [7], and the mitochondrial genome of *D. pteronyssinus *has recently been sequenced [8].

The *Ixodes scapularis *(black-legged tick) genome sequencing project marked the beginning of the genomics era for the field of acarology. As previously observed, there appears to be no patterns regarding genome size in the Acari. Flow cytometry based genome estimates indicated a large haploid genome for all Ixodida with a mean of 1281 Mbp (approx. 1.31 pg) for the Argasidae and 2671 Mbp (approx. 2.73 pg) for the Ixodidae [9]. An exciting development for mite genomics has been the publication of a preliminary genome survey for the honey-bee mite *Varroa destructor *[10]. At 565 Mbp, the *Varroa *genome is larger than many insects, including its host *Apis melifera *(262 Mbp). In contrast, the two-spotted spider mite *Tetranychus urticae *is predicted to possess a genome of only 75 Mb (0.08 pg) [11], however, this estimate was recently revised upward to 90.7 Mbp [12]. A similarly small nuclear genome of 88-90 Mbp was estimated in the phytoseiid mite *Metaseiulus occidentalis *[13], with the orbatid mite *Archegozetes longisetosus *genome estimated to be 150 Mbp [12].

Attempts to estimate the *S. scabiei *genome size accurately using flow cytometry have been impeded by the inability to obtain sufficient cell numbers from egg and whole-body preparations. The nuclei are very small in comparison to nuclei from mosquito cell lines, suggesting that the scabies genome may also be small (Fischer, unpublished observations). Similar issues were reported in *M. occidentalis*, where flow cytometry failed to resolve genome size, with estimates ranging from 35-160 Mbp, depending on egg age [13]. Due to the small size of mites, tissue dissections are difficult, hindering aquisition of homogenous cell preparations. An alternative approach utlilising quantitative-PCR (qPCR) was suggested to be particularly useful for organisms where the genome size is expected to be small and the availability of genetic material limited [13]. Based on on a pioneering study by Wilhelm [14], this method has proven reliable for a number of species, including *Saccharomyces cerevisiae, Xiphorphours maculaus, Homo sapiens *[14], *Musca domestica*, and *Drosophila melanogaster *[15]. Here, we use qPCR to estimate the genome sizes of *S. scabiei, P. ovis *and *D. pteronyssinus*, with the purpose of informing future sequencing projects. Additionally, the chromosome number of *S. scabiei *was determined, providing a starting point for understanding the genetic organisation and evolution for this species.

## Methods

### Source of samples

Scabies mites (*Sarcoptes scabiei *var. *suis) *were obtained from a colony maintained on pigs (*Sus scrofa*) at the Centre for Advanced Animal Studies (CAAS), University of Queensland, Gatton, QLD, Australia. Mites were isolated from heavily infested skin crusts as described previously [3]. Sheep scab mites (*Psoroptes ovis) *were harvested from infested donor lambs maintained at the Moredun Research Institute as previously described [16]. Ethical approvals for this work were obtained from the Queensland Institute of Medical Research and Queensland Department of Employment, Economic Development and Innovation, and the Moredun Research Institute Experiment Committees respectively. House dust mites (*Dermatophagoides pteronyssinus)*, separated into the two sexes, were purchased from the Siriraj Dust Mite Centre for Services and Research, Department of Parasitology, Mahidol University, Bangkok, Thailand. Isolated mites were stored at -80°C until further processing. The methods to separate sexes and adults from larvae and nymphs of the individual mite species are well established in the three laboratories involved. Adult female scabies and psoroptes mites are easy to distinguish from males due to their much larger size. The adult female scabies mites are approximately 500 μm long compared to the smaller approximately 250 μm long males. In addition adult scabies mite males can be separated from larvae and nymphs based on size, a darker, and more sclerotized cuticle and leg number. Mature female Psoroptes are 550-750 μm long, with a striate cuticle and four long and 16 short dorsal somatic setae [17,18] while males are about one-fourth smaller, and they have an additional, larger posterodorsal cuticular plate and a pair of posteroventral adanal suckers. Adult female house dust mites measure approximately the same length as males but are 0.32 mm in width compared to 2.4 mm wide males. Males are more sclerotized with enlarged legs I and III [19]

### DNA extraction

Genomic DNA (gDNA) preparations used for qPCR included: a) *S. scabiei *var. *suis *female mites (100) and mixed life-stage mites (~500); b) *P. ovis *female mites (75); c) *D. pteronyssinus *male (10 mg) and female (15 mg) mites; d) as a positive control for the method, *Pichia pastoris *strain *GS115 *gDNA was used.

Genomic DNA was extracted by homogenising mites using a motorised micropestle in a purpose-designed eppendorf tube (Kontes, Kindle Chase) over liquid-nitrogen. The homogenate was resuspended in 2 ml of digestion buffer (800 mM Guanidine HCl; 30 mM Tris-Cl, pH8.0; 30 mM EDTA, pH8.0; 5% Tween-20; 0.5%Triton X-100), supplemented with 2 μL RNase A (Qiagen, 20 mg/ml) and incubated at 37°C for 30 min. 100 μL proteinase K (Qiagen, 20 mg/ml) was added and incubated at 50°C for 1 hour with gentle agitation. The lysate was spun at 4000 g at 4°C for 10 min and the supernatant was loaded on to a Qiagen genomic tip 20/G of the Qiagen Blood & Cell Culture DNA Mini Kit (Qiagen, Doncaster, Australia). DNA extraction was performed according to manufacturer's instructions. Final DNA pellets were resuspended in 30-100 μL 15 mM Tris-EDTA Buffer and aliquots stored at -80°C. DNA concentration and purity was measured by a) measuring absorbance at 260 nm using the ND-1000 Spectrophotometer (Thermo Scientific, Wilmington, USA), b) Qubit-IT pico green HS dsDNA reagent (Invitrogen, Mulgrave, Australia) in a fluorometer (Biotek Synergy-4) and c) agarose gel electrophoresis with ethidium bromide staining. Only DNA samples for which the three quantification methods concurred were used for subsequent qPCR.

### PCR primer design and preparation of qPCR standards

Based on their successful use in similar studies [13], and availability of sequence information, two single copy nuclear genes were selected for this study--actin and elongation factor 1α (EF1α). Primers were designed from EST sequence available for *S. scabiei *actin [EU624346], EF1α [JQ236667]; *Psoroptes ovis *actin [BQ834989], EF1 α [BQ835070]; *D. pteronyssinus *EF1α [EU152830]. For positive control experiments PCR primers were also designed for *P. pastoris *actin [AF216956] and elongation factor 3 (EF3) [FN392322] (Table 1).

**Table 1:** Primers used in this study

Gene	Primer	Sequence (5'-3')	Size
Actin (*S. scabiei*)	SsAct F	CAACCATCCTTCTTGGGTATG	311 bp
	SsAct R	CCAGCTTCGTCGTATTCTTGT	
Actin (*P. ovis)*	PoAct F	CATCAAGGTGTCATGGTTGG	225 bp
	PoAct R	GGCTTTTGGATTCAATGGAG	
Actin (*P. pastoris*)	PpAct F	AGCGCGCCCATTTCTTACCGT	203 bp
	PpAct R	TCTCCGGCGTATCCGGCCTT	
EF1a (*S. scabiei*)	SsEF1a F	TTGGCTTATACCTTGGGTGTG	181 bp
	SsEF1a R	CACCGTTCCATCCAGAGATT	
EF1a (*D. pteronyssius)*	DpEF1a F	CCCGTGAACATGCTTTGCTTGCC	175 bp
	DpEF1a R	ACAAAAGCGACGGTAGCCGGA	
EF1a (*P. ovis)*	PoEF1a F	CAAATATGCCTGGGTTTTGG	205 bp
	PoEF1a R	TTCACCAGTACCAGCAGCAA	
EF3 (*P. pastoris*)	PpEF3 F	GTCGCCATTTTGGATGCTAT	251 bp
	PpEF3 R	AGGGTGTGGACAGTTTCTGG	

To ensure primer and PCR conditions were optimal, that the PCR was specific, and to prepare plasmid standards, a series of validation PCRs were run prior to commencing qPCR for each mite species and primer combination. Firstly, gradient PCRs were performed to determine optimum annealing temperatures. Reactions contained 0.2 mM dNTPs, 0.4 μM of each primer, 2.5 mM MgCl_2 _, 0.2 U Taq Polymerase (AmpliTaq Gold, Applied Biosystems), and 1 μL template gDNA. Cycling times were 95°C for 10 minutes, followed by 30 cycles of 94°C for 15 s, 55-65°C for 30 s, and 72°C for 30 s. Products were visualised on 1.5% agarose gels, purified (QiaQuick purification kit, Qiagen) and cloned into pGEM-T vectors (Promega, Alexandria, Australia). After sequencing to confirm product specificity, plasmids were linearised with *NotI*, purified and quantified as described previously.

### Quantitative PCR

Quantitative real-time PCR was performed using Sensimix SYBR green (Bioline, Alexandria, Australia) in a Rotorgene 6000 cycler (Qiagen). Reactions contain 1× SYBR green mastermix, 0.4 μM of each primer, and 1 μL template DNA. Each run consisted of a series of linearised plasmid standards (seven, 10-fold dilutions), genomic DNA and a no-template control, with all reactions run in duplicate. Copy number of standards was calculated using a DNA weight to moles calculator (http://molbiol.edu.ru/eng/scripts/h01_07.html), which determined the number of copies based on the concentration and size of the plasmid. A standard curve was generated from the Ct values of the standards using Rotorgene software, which was used to calculate the copy number of the unknown gDNA. Only runs with a standard curve efficiency > 90% and < 0.5 Ct standard deviation of duplicates were utilised for calculation of genome estimates.

The weight of a single copy of the nuclear genome (C-value) in picograms was obtained by dividing the input template concentration by the qPCR derived copy number. The estimated genome size of the unknowns was calculated using the formula Genome size (bp) = (0.978 × 10^9^) × C (pg). This is based on the formula of Dolezel [20], where the mean weight of one nucleotide base pair = 1.023 × 10^-9 ^pg.

### Determining the chromosome number in *Sarcoptes scabiei*

Female *S. scabiei *mites were isolated from infested pig skin and placed on glass petri dishes. Eggs that were deposited within a time frame of a few hours were processed, since they were most likely alive and provide dividing cells.

Superfrost slides (Manzel-Glaser, Braunschweig, Germany) that had been cleaned in 100% ethanol were dried and two eggs per slide were placed 2 cm apart on the slide surface. To dissolve the outer chorion, eggs were bathed in 10 μL 1% sodium hypochlorite in dH_2_0 for 5 minutes. This solution was removed and replaced with 10 μL 1% colchicine (Sigma) in 0.7% sodium chloride/10 mM Tris pH 7.5. Eggs were incubated in the dark for 30 minutes in a humidity chamber, the colchicine solution was removed and replaced by 20 μL 0.075 M potassium chloride for 15 minutes. The hypotonic solution was removed and 20 μL fixative (3:1 methanol:acetic acid) added. A cover slip was placed on the slide and the eggs were squashed using the flat end of a pencil. The slide was placed on dry-ice for several minutes before the coverslip was removed. The slides and coverslip were air-dried before the addition of DAPI stain (ProLong Gold, Invitrogen). The cover slip was replaced, and the slides were viewed with the DeltaVision deconvolution microscope. The data generated through deconvolution (SoftWORx software) was used to generate a 360° rotational image of the chromosomal spread. Two people assessed the number of chromosomes in 18 eggs which had multiple cells in metaphase.

## Results and Discussion

### qPCR based genome estimation

qPCR on the positive control *P. pastoris *gDNA with actin and EF3 primers yielded a mean genome estimate of 8.7 **± **0.5 Mbp (Table 2), within 10% of the actual size of 9.43 Mbp [21], attesting to the validity of this technique.

**Table 2:** qPCR based genome estimates of *S. scabiei, P. ovis *and D. pteronyssius

Species	Sex	Gene	n	C (mean, ± SD, pg)	Genome size (mean, ± SD, Mbp)
***S.scabiei***	Female	Actin	2	0.095 (± 0.042)	93 (± 41)
		EF1a	3	0.091 (± 0.005)	89 (± 14)
	Mixed	Actin	1	0.107	105
		EF1a	2	0.101 (± 0.014)	98 (± 14)
Average				**0.099 (**± **0.007)**	**96 (± 7)**

***P. ovis***	Female	Actin	2	0.089 (± 0.001)	87 **± **(0.5)
		EF1a	3	0.086 (± 0.008)	84 (**± **8)
Average				**0.088 (± 0.002)**	**86 (± 2)**

***D. pteronyssinus***	Female	EF1a	2	0.155 (± 0.0004)	151 (**± **0.4)
	Male	EF1a	2	0.223 (± 0.004)	218 (± 4)

***P. pastoris ***		EF3	2	0.009 (0.0004)	8.37 (± 0.4)
		Actin	2	0.009 (± 0.001)	9.05 (± 0.6)
Average				**0.009**	**8.71 (**± **0.5)**

qPCR of two single-copy genes for two gDNA preparations of *S. scabiei *gave a mean genome estimate of 96 ± 7 Mbp (Table 2). There was no large difference overall between estimates using the different primer pairs, although the actin PCR gave a highly variable result with female mites, with variation in the EF1α PCRs lower. We were unable to sample male-only preparations due to insufficient numbers, but genome estimates of mixed life-stage gDNA were not significantly different to female only, an observation that agrees with previous suggestions that haplodiploidy or other forms of polyploidy is not a feature of *Sarcoptes *[22].

The genome size estimate for *P.ovis *female mites was smaller than *S. scabiei*, at 86 ± 2 Mbp. These estimates are very close to that of *M. occidentalis *(88-90 Mbp) and *T. urticae *(75-90 Mbp), collectively representing the smallest known arthropod genomes to date.

Interestingly, the genome of *D. pteronyssinus *is predicted to be larger than its parasitic relatives, at 151 Mbp for females and 218 Mbp for males (Table 2). The reason for the larger size estimate in males is not understood, and could be reflective of low sample numbers or different developmental stages. As the chromosome number, sex determination system and karyotype have not been determined for house dust mites, it is difficult to speculate without further investigation.

Although flow cytometry remains the "gold standard" for determination of genome size in the absence of sequencing, here we confirm the usefulness of the qPCR method, especially where genetic material is limited. While a limitation of the method is its sensitivity to inaccuracies in DNA concentration and PCR efficiency, it is a quick and useful way to establish general estimates of genome size where more accurate flow cytometry may not be possible.

### Chromosome number in *S. scabiei*

**Figure 1:**
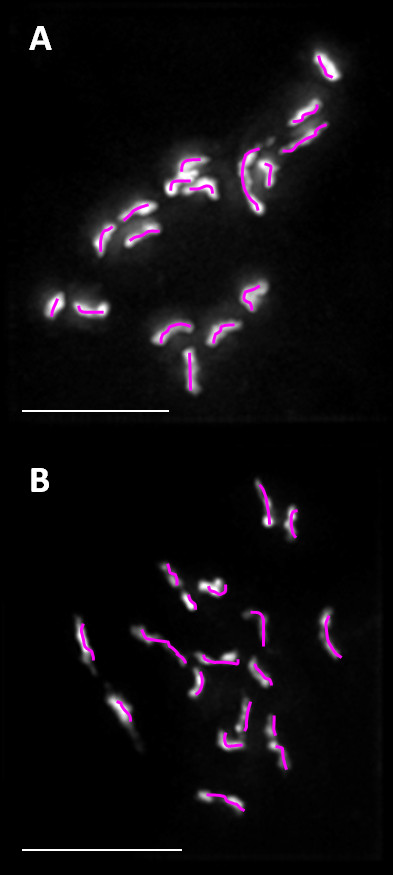
**Chromosomal spreads of single *Sarcoptes scabiei *cells**. A) 17 chromosomes can be counted while B) shows 18 chromosomes. Chromosomes are highlighted in pink for clarification. Scale bar = 5 μm.

Using 360° rotational images of chromosomal spreads from *S. scabiei *eggs, the chromosome number of embryonic cells from a single egg was either 17 or 18 very small chromosomes, of which the largest chromosome was < 2.5 μm (Figure 1). These findings accord with the qPCR results indicating a small scabies mite genome. While the cause for the disparate numbers is unknown, it may arise because of an XO sex determination mechanism, where males lack the sex chromosome and therefore have one less chromosome than the female. This occurs in a range of Ecdysozoan invertebrates including nematodes and arthropods, and has been demonstrated in the Acaridae [23]. These results again support the absence of haplodiploidy, where haploid males develop from unfertilised eggs and diploid females develop from fertilised eggs. Haplodiploidy in *S. scabiei *was also excluded in a previous study [22], as heterogeneity was demonstrated in microsatellite genotypes from male mites. Previous surveys of the Astigmata have demonstrated the prevailing sex determining mechanism to be diplo-diploidly, with chromosome numbers ranging from 10-18 [24,25].

## Conclusions

There appears to be little correlation between genome size, chromosome number, organism complexity, or phylogenetic relatedness, commonly referred to as the "C-value enigma" [26]. However, key factors influencing small genome sizes may include small cell size (positively correlated), developmental complexity (negatively correlated), and maintenance or loss of non-coding DNA [27].

The smallest arthropod genome sequenced to date is that of the ectoparasitic body louse, *Pediculus humanus humanus *[28]. At 108 Mb, the body louse shows a reduced, but markedly functional genome. Relative to other sequenced arthropods, the most marked reductions were seen in gene families associated with environmental interaction [28], and thus it is has been postulated that parasitic species may have smaller genomes than their non-parasitic counterparts, as an adaptation to parasitism and ecological niche leads to the expansion and contraction of gene families [28,29]. Our current results accord with this hypothesis, as the free-living house dust mite genome was estimated to be larger than that of the parasitic *Sarcoptes *or *Psoroptes *mites. In contrast, the genome of *V. destructor*, also parasitic, is considerably larger at 565 Mb [10]. Interestingly *S. scabiei *has been shown to contain a multi-gene family of inactivated protease paralogues (SMIPPs), which to date have not been identified in either house dust mite or *P. ovis *[30,31]. It has been recently shown that these proteins can inhibit the human complement system, suggesting this gene family expansion is a specific adaptation to burrowing and immune evasion [32].

The degree of non-coding and repetitive DNA, intron size and transposable elements also appears to play a role in genome size. Ticks for example have up to 70% of their genome as non-coding/repetitive [9]. Conversely, the body louse genome has less than 1% of their genome devoted to transposable elements and introns, considerably less than *Drosophila *[28]. It has been suggested that transposable elements cannot be established in eukaryotic genomes < 100 Mb [33], so it will be very interesting to see whether these elements are present in the mites studied here. The majority of sequence data obtained to date from *S. scabiei *has been from cDNA, and only very few genome sequences are published. We have to date identified genomic sequences of 12 genes (7 complete, 5 incomplete) containing a total of 25 introns. The introns in all but one of the genes were all ≤ 100 bp with an average size of 69 bp for these 13 introns. Of the 11 introns in the remaining gene (encoding a large transmembrane protein), 7 were ≤ 100 bp with the remaining 4 introns being 547 bp , 248 bp , 560 bp and 381 bp . The average size of all the 25 different introns we have identified to date is 128 bp . Interestingly the average intron size in the *V. destructor *sodium channel gene is 2.7 kb [34], compared to only 203 bp in the partial *S. scabiei *gene sequence (domains II-IV)[35]. Altogether, this data supports the estimation of a very small genome in *Sarcoptes*.

Sequencing of the *Sarcoptes scabiei *genome is scheduled to commence in the near future, and we encourage the extension of efforts to also include *D. pteronyssinus *and *P. ovis*. A comparative genomic approach between these three skin-feeding mites is likely to highlight fascinating genetic similarities and differences between parasitic and free-living mites, and burrowing versus non-burrowing parasitic mites, and to provide essential information to inform control strategies for these important species. This would also be complemented by comparison with the recently sequenced *P. humanus *and *V. destructor *genomes. In this study, we have confirmed the small size of these mite genomes, which augurs well for the successful completion of future sequencing projects.

## Competing interests

'The authors declare that they have no competing interests

## Authors' contributions

KM conceived the study, carried out the cloning, qPCR and analysis, and drafted the manuscript. CW carried out the chromosomal studies. STGB provided *P. ovis *and participated in design of the study. DH carried out bioinformatic analysis, participated in study design and helped to draft the manuscript. JMc provided mites and helped to draft the manuscript. KF participated in design and coordination of the study, provided mites and carried out DNA extractions, all authors read and approved the final manuscript.
